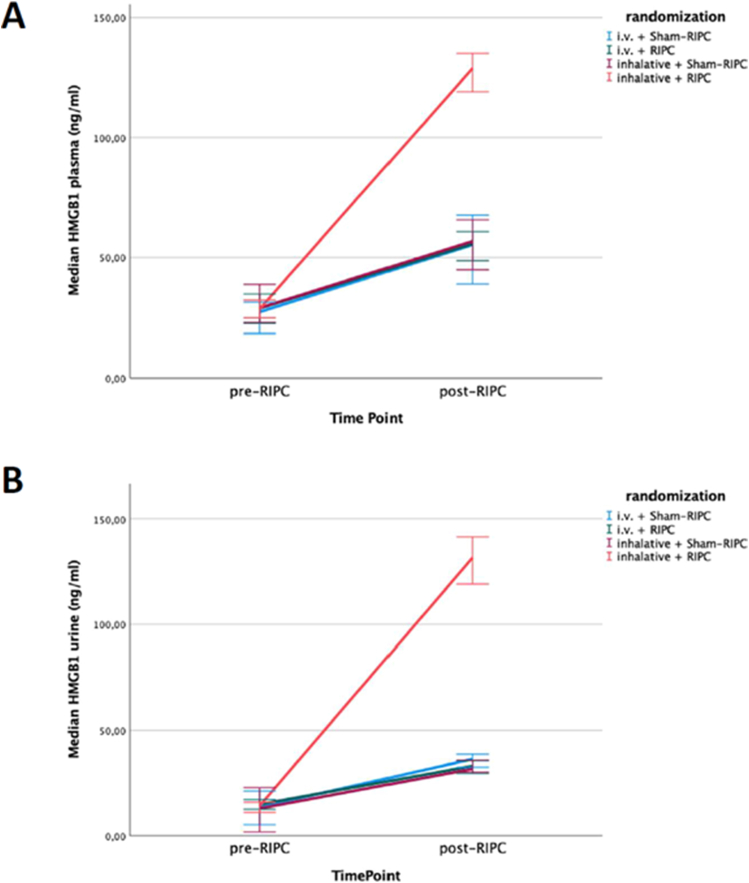# Impact of propofol or sevoflurane on the renoprotective effect of remote ischaemic preconditioning in cardiac surgery: the HypnoRenalRIP randomised clinical trial

**DOI:** 10.1016/j.bja.2025.08.055

**Published:** 2025-09-22

**Authors:** Alexander Zarbock, Ludwig Maximilian Schöne, John A. Kellum, Joachim Gerss, Raphael Weiss, Hendrik Booke, Melanie Meersch

**Affiliations:** 1 Department of Anaesthesiology, Intensive Care Medicine and Pain Medicine, University Hospital Münster, Münster, Germany; 2 Department of Anesthesiology, Critical Care and Pain Medicine, The University of Texas Health Science Center at Houston, Houston, TX, USA; 3 Center for Critical Care Nephrology, Department of Critical Care Medicine, University of Pittsburgh, Pittsburgh, PA, USA; 4 Institute of Biostatistics and Clinical Research, University of Münster, Münster, Germany

**Keywords:** acute kidney injury, biomarker, cardiac surgery, insulin-like growth factor-binding protein 7, propofol, remote ischaemic preconditioning, tissue inhibitor of metalloproteinases-2

## Abstract

**Background:**

Remote ischaemic preconditioning (RIPC) might reduce acute kidney injury after cardiac surgery. Protective effects appear to be restricted to patients with early and transient increases in two cell cycle arrest markers, tissue inhibitor of metalloproteinases-2 (TIMP-2) and insulin-like growth factor-binding protein 7 (IGFBP7), in urine. Studies suggest that propofol can attenuate the preconditioning effect on the myocardium. This study investigated whether propofol diminishes the renoprotective effect associated with the early transient increases in TIMP-2 and IGFBP7.

**Methods:**

This was a single-centre, prospective randomised double-blind 2×2 factorial clinical trial of high-risk patients undergoing cardiac surgery. Patients were randomised to receive either propofol+sham-RIPC, propofol+RIPC, sevoflurane+sham-RIPC, or sevoflurane+RIPC. The primary outcome measure was the change in the product of urinary concentrations of TIMP-2 and IGFBP7 ([TIMP-2]·[IGFBP7]) from before to after the intervention.

**Results:**

We enrolled 160 participants in the trial (propofol+sham-RIPC: *n*=20, propofol+RIPC: *n*=60, sevoflurane+sham-RIPC: *n*=20, sevoflurane+RIPC: *n*=60). The median change in [TIMP-2]·[IGFBP7] as an indicator of response to RIPC was greater in participants receiving sevoflurane (0.070; interquartile range, −0.120 to 0.418) compared with those receiving propofol (−0.015; interquartile range, −0.138 to 0.068; *P*=0.022). Conversely, elevated [TIMP-2]·[IGFBP7] as a sign of renal stress in response to surgery was detected in all groups except for sevoflurane+RIPC (*P*=0.001). There were no statistically significant differences in patient-centred outcomes between groups.

**Conclusions:**

Transient increases in [TIMP-2]·[IGFBP7] induced by RIPC, which were associated with renoprotective effects, were only seen with sevoflurane anaesthesia, but not when propofol was used. The association of biomarker concentrations and choice of anaesthetic agent suggests that propofol can attenuate the renoprotective effects of remote ischaemic preconditioning.

**Clinical trial registration:**

DRKS00014989

**Editor’s key points**

• Remote ischaemic preconditioning (RIPC) has shown promising organ-protective properties in various experimental models, but its clinical applications are unclear.
• The authors analysed the impact of propofol or sevoflurane anaesthesia on RIPC in reducing acute kidney injury (AKI) after cardiac surgery as reflected in the cell cycle arrest biomarkers TIMP-2 and IGFBP7 in urine.
• The median increase in the urine biomarkers TIMP-2 and IGFBP7 as an indicator of response to RIPC was greater in the sevoflurane group compared with the propofol group.
• These findings suggest that propofol can attenuate the renoprotective effects of RIPC and should be avoided in future studies of RIPC impact on organ protection.

Acute kidney injury (AKI) is a common and potentially severe complication after cardiac surgery, especially when cardiopulmonary bypass (CPB) is used. Up to 30% of patients undergoing cardiac surgery develop AKI when defined using changes in serum creatinine,^1^ and as many as 80% when urine output is also considered.^2^ AKI is associated with complications including delirium, infection, bleeding, chronic kidney disease, chronic dialysis dependency, cardiovascular disease, and death, and it is subsequently associated with increased health care resource utilisation and costs.^3^, ^4^, ^5^, ^6^, ^7^, ^8^ This illustrates the urgent, currently unmet need for preventive options.

Remote ischaemic preconditioning (RIPC) is a simple and safe procedure that consists of brief episodes of ischaemia and reperfusion of remote tissue before a subsequent injury to the target organ occurs. We have previously shown that RIPC successfully reduced the occurrence of AKI after cardiac surgery in high-risk patients, and that the effectiveness of this intervention was dependent on the transient release of two kidney cell cycle arrest biomarkers immediately after the procedure: tissue inhibitor of metalloproteinases-2 and insulin-like growth factor-binding protein 7 ([TIMP-2]·[IGFBP7]).^9^ The production of [TIMP-2]·[IGFBP7] is mediated by high mobility group box protein-1 (HMGB1), a damage-associated pattern, released by RIPC.^10^ For detection of renal stress, the product of [TIMP-2] and [IGFBP7] ([TIMP-2]·[IGFBP7]) is used. Patients with urinary [TIMP-2]·[IGFBP7] ≥0.5 ng ml^−2^ 1000^−1^ after the RIPC intervention and before surgery had a significantly reduced rate of AKI compared with patients with a lower urinary [TIMP-2]·[IGFBP7] concentration, and the biomarker concentrations after surgery predicted subsequent development of AKI.^9^ Two large randomised trials enrolling lower-risk patients did not show a reduction of AKI by RIPC.^11^^,^^12^ However, most patients in both trials received propofol as a core component of their anaesthetic technique. Several experimental studies have suggested that propofol can mitigate the beneficial effects of RIPC. Subsequent work has also suggested that this might also be the case in human subjects.^13^, ^14^, ^15^

Given the uncertainty regarding the interference of anaesthetic agents on the renoprotective effects of RIPC, we performed a single-centre randomised clinical trial primarily focusing on the effect of RIPC-induced release of TIMP-2 and IGFBP7 as a measurable surrogate for the induction of renoprotective mechanisms. The purpose of this study was to investigate whether propofol diminishes the renoprotective effect of RIPC. We hypothesised that RIPC has an effect on [TIMP-2]·[IGFBP7] in sevoflurane-anaesthetised patients, but not in propofol-anaesthetised patients.

## Methods

### Study design and review board approval

This study was conducted as a single-centre, prospective, randomised, double-blind, 2×2 factorial clinical trial. Institutional review board approval was obtained from the research ethics committee of the chamber of physicians Westfalen-Lippe and the University of Münster (2018-170-f-S). The study was conducted in accordance with the Declaration of Helsinki (Fortaleza, 2013). Written informed consent was obtained from all participating patients following local requirements. Study design and preparation of the manuscript followed the Consolidated Standards of Reporting Trials (CONSORT) statement recommendations. The study was registered at www.drks.de (DRKS00014989).

### Participants

Eligible patients were adults at high risk for AKI who underwent cardiac surgery with the use of CPB at the University Hospital of Muenster between June 2018 and November 2020. The study ended with the last follow-up in February 2021. A Cleveland Score of 6 or higher was used to define patients at high risk for AKI.^16^ The score is composed of different risk factors, including patient characteristics, comorbidities, and type of surgery.^16^

Exclusion criteria were myocardial infarction within 7 days preceding surgery, off-pump cardiac surgery or preexisting cardiac assist devices, preexisting AKI (stage 1 or higher), chronic kidney disease with estimated glomerular filtration rate (eGFR) <30 ml min^−1^ 1.73 m^−2^ (or dialysis dependent kidney disease), kidney transplant within the last 12 months, peripheral artery disease in both upper extremities, pregnancy, breastfeeding and women of childbearing potential, hepatic insufficiency or existing hepatorenal syndrome, use of sulfonamides (including thiazide diuretics) within 7 days before surgery (preconditioning-blocking medication), participation in another interventional trial in the last 3 months, persons with any kind of dependency on the investigator or employed by the sponsor or investigator, and persons held in an institution by legal or official order.

### Randomisation

Participants were randomised on a 1:3:1:3 basis without stratification into one of the four groups: (1) propofol+sham-RIPC, (2) propofol+RIPC, (3) sevoflurane+sham-RIPC, and (4) sevoflurane+RIPC. Randomisation codes were computer generated and concealed from investigators. Allocation was performed by an independent investigator who was not involved in either the clinical care of trial participants or in the application of the intervention. Data collectors, blinded outcome adjudicators, and analysts were completely independent of the care of the patients, as was the physician who applied the intervention.

### Procedures

Participants randomised to the propofol group received propofol for anaesthesia induction (0.5–1.0 mg kg^−1^ demand-adapted) and maintenance (3–4 mg kg^−1^ h^−1^). Participants randomised to the sevoflurane group received thiopental (3.0 mg kg^−1^) for anaesthesia induction and sevoflurane (0.8–1.5 vol% demand-adapted) for maintenance. In addition, sufentanil was administered as analgesic (0.7 μg kg^−1^ bolus and 1.0 μg kg^−1^ h^−1^ maintenance) and cis-atracurium for neuromuscular block (0.2 mg kg^−1^) according to the standards of care at our centre. RIPC was performed after anaesthesia induction and before skin incision through a standard manual blood pressure cuff (Flexiport^TM^ reusable blood pressure cuff, Welch Allyn®, Skaneateles Falls, NY, USA) inflation to an upper arm with three cycles of 5 min cuff inflation to 200 or 50 mm Hg higher than systolic pressure (ischaemia) followed by 5 min cuff deflation (reperfusion). In the sham-RIPC group, patients received three cycles of 5 min cuff inflation to 20 mm Hg followed by 5 min cuff deflation. The treating clinicians were blinded to the intervention at all times, and RIPC or sham was performed by an independent member of the study group not involved in the treatment of the patient or the data analysis. The surgical procedure and perioperative care were performed according to the standards of the centre.

### Outcomes

Changes in [TIMP-2]·[IGFBP7] between before and after the intervention in participants receiving RIPC were the primary endpoint of this study. This endpoint was chosen because it was shown that a transient increase of [TIMP-2]·[IGFBP7] is necessary for RIPC-induced renoprotection.^9^ In the prespecified primary statistical analysis, the randomised group 4 (sevoflurane+RIPC) is compared with group 2 (propofol+RIPC). In further prespecified statistical analyses, the primary endpoint in the randomised group 2 (propofol+RIPC) is compared with group 1 (propofol+sham-RIPC), and group 4 (sevoflurane+RIPC) is compared with group 3 (sevoflurane+sham-RIPC). Secondary endpoints included the incidence and severity of AKI within 72 h after surgery according to the KDIGO guidelines,^17^ renal recovery (defined as serum creatinine <0.5 mg dl^−1^ higher than baseline) at 30 and 90 days, dialysis dependency at 30 and 90 days, mortality at 30 and 90 days, and major adverse kidney events (MAKE) at 30 and 90 days (combined endpoint consisting of persistent renal dysfunction [serum creatinine ≥0.5 mg dl^−1^ compared with baseline], dialysis dependency, and mortality). In *post hoc* analysis, HMGB-1 concentrations before and after the intervention, [TIMP-2]·[IGFBP7] concentration 4 and 12 h after CPB, hospital length of stay, ICU length of stay, and potential adverse events of RIPC (perioperative myocardial infarction, cerebrovascular events or stroke, pulmonary embolism, residual adverse effects on corresponding limb, and petechial bleeding) were analysed.

### Data and sample collection

Clinical variables were extracted from medical records. Samples were collected at four time points: pre-RIPC, 45 min after RIPC, 4 h after CPB, and 12 h after CPB. The time points for the measurements of [TIMP-2]·[IGFBP7] were chosen based on previous publications.^9^^,^^18^ Urinary [TIMP-2]·[IGFBP7] levels were measured using the NephroCheck® test (Astute Medical, San Diego, CA, USA/Biomérieux, Lyon, France). HMGB-1 was measured with a commercially available assay^19^ according to the manufacturer’s protocol in plasma and urine samples at the time points before and after RIPC.

### Statistical analysis

We calculated a necessary sample size of 160 participants based on the primary endpoint and the prespecified primary statistical analysis. The following primary endpoint data were expected: change in [TIMP-2]·[IGFBP7] post-RIPC minus pre-RIPC. Propofol+control (sham-RIPC) (*n*=20): *μ*_1_=−0.0140 with standard deviation *s*_1_=0.50842, propofol+RIPC (*n*=60): *μ*_2_=−0.0140 with standard deviation *s*_2_=0.50842, sevoflurane+control (sham-RIPC) (*n*=20): *μ*_3_=−0.0140 with standard deviation *s*_3_=0.50842 (based on published data^9^); and sevoflurane+RIPC (*n*=60): *μ*_4_=0.3088 with standard deviation *s*_4_=0.85328 (based on published data^9^). The expected means and standard deviations in groups 1 and 2 (*μ*_1_, *s*_1_, *μ*_2_, *s*_2_) result from the basic hypothesis that in propofol-anaesthetised patients, RIPC has the same (null) effect as observed in the sham-RIPC group of our previous trial.^9^ Under the above assumptions and with the above numbers of participants, in the primary statistical analysis, the primary hypothesis H_0_: *μ*_2(propofol+RIPC)_=*μ*_4(sevoflurane+RIPC)_ can be rejected with 75.3% power. Sample size calculation was performed based on simulated data with 10 000 simulation runs.

Categorical variables were summarised by frequency tables. Treatment groups were compared with the χ^2^ test (or Fisher’s exact test if the respective frequency tables contained cells with expected counts <5). Normally distributed continuous variables were expressed as mean (standard deviation), and were compared between groups with unpaired Student’s *t-*test. Continuous variables, which were not normally distributed, were analysed using nonparametric tests (Kruskal–Wallis test, Mann–Whitney *U* test). Statistical analysis of the primary endpoint was performed using a quantile (median) regression model with the independent variable [TIMP-2]·[IGFBP7] pre-RIPC, main effects of the factors RIPC (*vs* sham-RIPC) and anaesthetic agent (sevoflurane *vs* propofol), and its interaction. In exploratory analyses, two-sided *P*-values were considered significant for *P*≤0.05. Statistical analyses were performed using SPSS software (version 27 for Windows, Redmond, WA, USA) and SAS (version 9.4 for Windows, SAS Institute Inc., Cary, NC, USA).

## Results

### Participants

**Fig 1:**
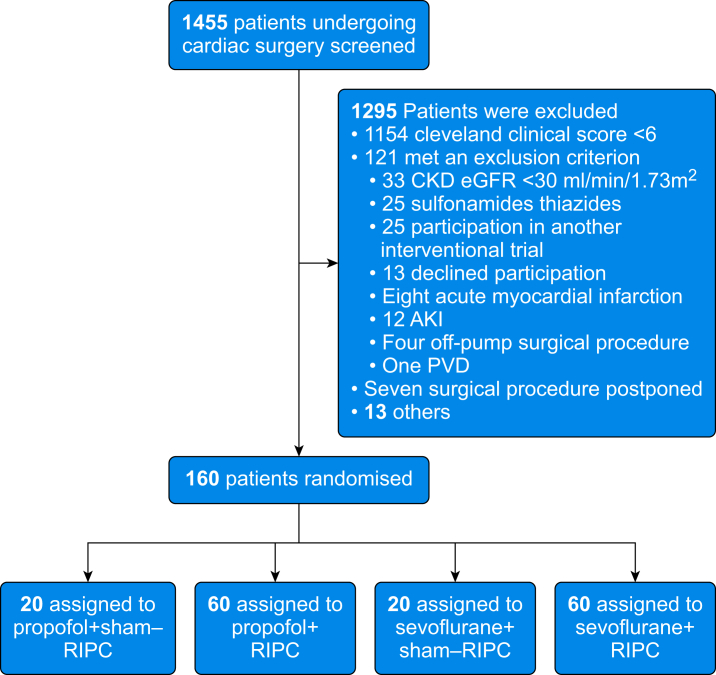
Participant flow. Enrolled participants were randomised to four groups. AKI, acute kidney injury; CKD, chronic kidney disease; eGFR, estimated glomerular filtration rate; PVD, peripheral vascular disease; RIPC, remote ischaemic preconditioning.

Of 1457 patients screened for the trial, 160 were included in the HypnoRenalRIP trial (Fig. 1). All participants received the allocated treatment and were included in the primary analysis. Participant characteristics and operative data were comparable between the groups (Table 1). Three patients were lost to follow-up after 90 days.

**Table 1:** Participant characteristics and operative data. ACEi, angiotensin converting enzyme inhibitor; ARB, angiotensin receptor blocker; COPD, chronic obstructive pulmonary disease; CPB, cardiopulmonary bypass; EF, ejection fraction; RIPC, remote ischaemic preconditioning.

	Propofol+sham-RIPC	Propofol+RIPC	Sevoflurane+sham-RIPC	Sevoflurane+RIPC
	(*n*=20)	(*n*=60)	(*n*=20)	(*n*=60)
Age, mean (range) (yr)	74.7 (59–85)	69.7 (34–86)	67.0 (27–85)	71.3 (48–86)
Sex (male), *n* (%)	12 (60.0)	43 (71.7)	11 (55.0)	37 (61.7)
Serum creatinine, mean (sd) (mg dl^−1^)	1.2 (0.3)	1.2 (0.3)	1.2 (0.3)	1.2 (0.3)
ASA physical status, *n* (%)				
1	0	0	0	1 (1.7)
2	2 (10.0)	8 (13.3)	2 (10.0)	17 (10.6)
3	15 (75.0)	46 (76.7)	15 (75.0)	52 (86.7)
4	3 (15.0)	6 (10.0)	3 (15.0)	2 (3.3)
Comorbidities, *n* (%)				
Hypertension	15 (75.0)	43 (71.7)	16 (80.0)	53 (88.3)
Congestive heart failure	17 (85.0)	47 (78.3)	18 (90.0)	48 (80.0)
Diabetes mellitus	8 (40.0)	16 (26.7)	5 (25.0)	20 (33.3)
COPD	4 (20.0)	15 (25.0)	1 (5.0)	11 (18.3)
Chronic kidney disease	7 (35.0)	16 (26.7)	4 (20.0)	17 (28.3)
Previous heart surgery	5 (25.0)	16 (26.7)	5 (25.0)	25 (41.7)
Left ventricular EF <35%	3 (15.0)	4 (6.7)	3 (15.0)	6 (10.0)
EuroSCORE, mean (sd)	3.7 (1.8)	3.2 (2.2)	2.9 (2.3)	3.4 (2.3)
Medication, *n* (%)				
Aspirin	6 (30.0)	27 (45.0)	9 (45.0)	20 (33.3)
Clopidogrel	2 (10.0)	3 (5.0)	1 (5.0)	1 (1.7)
Statins	14 (70.0)	37 (61.7)	9 (45.0)	35 (58.3)
ß blockers	15 (75.0)	43 (71.7)	16 (80.0)	43 (71.7)
ACEi/ARBs	13 (65.0)	41 (68.3)	17 (85.0)	45 (75.0)
Operative times, median (Q1–Q3) (min)				
CPB	149.0 (97.8–219.0)	129.5 (109.3–161.0)	152.0 (92.3–226.5)	135.0 (116.3–176.5)
Cross-clamp	107.0 (70.0–140.0)	89.0 (70.0–106.0)	94.5 (68.5–151.5)	97.0 (78.0–120.0)

### Primary analysis

Absolute changes in [TIMP-2]·[IGFBP7] were highest in the sevoflurane+RIPC group with a median increase of 0.070 (−0.120 to 0.418), and differed from the propofol+RIPC group where the change was −0.015 (−0.138 to 0.068) (*P*=0.022). Absolute changes also differed from the sevoflurane+sham-RIPC group, 0.005 (−0.455 to 0.053) (*P*=0.037). A difference to the propofol+sham-RIPC group did not achieve statistical significance (*P*=0.161) (Table 2). In sevoflurane-anaesthetised participants, the difference between the RIPC and sham-RIPC groups was significant (*P*=0.037), whereas in propofol-anaesthetised patients, the corresponding difference was not significant (*P*=0.755). All *P*-values were derived from the fitted quantile (median) regression model (Supplementary material).

**Table 2:** Tissue inhibitor of metalloproteinases-2 and insulin-like growth factor-binding protein 7 concentrations: changes in [TIMP-2]·[IGFBP7] for various groups. RIPC, remote ischaemic preconditioning; [TIMP-2]·[IGFBP7], log of product of tissue inhibitor of metalloproteinases-2, and insulin-like growth factor-binding protein 7 concentrations in X units. ∗*P*-values from pairwise comparisons of individual treatment groups with the sevoflurane+RIPC group (postulated as the most effective group).

[TIMP-2]·[IGFBP7]	Propofol+sham-RIPC	Propofol+RIPC	Sevoflurane+sham-RIPC	Sevoflurane+RIPC
	(*n*=20)	(*n*=60)	(*n*=20)	(*n*=60)
Absolute change of [TIMP-2]·[IGFBP7] (post-intervention−pre-intervention)				
Median (Q1–Q3)	0.025 (−0.245 to 0.088)	−0.015 (−0.138 to 0.068)	0.005 (−0.455 to 0.053)	0.070 (−0.120 to 0.418)
*P*-value∗	0.161	0.022	0.037	

**Fig 2:**
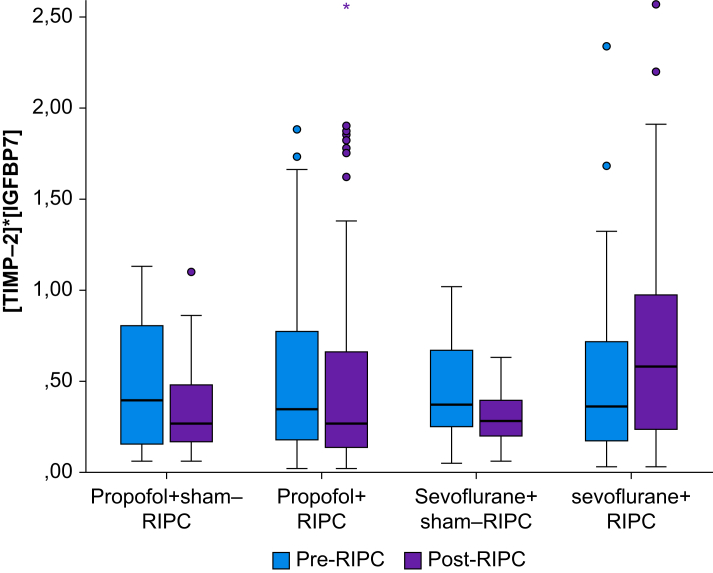
Product of TIMP-2 and IGFBP7 concentrations before (blue boxplots) and after (purple boxplots) the RIPC intervention in the four different groups. The post-RIPC measurements were taken 45 minutes after the intervention. RIPC, remote ischaemic preconditioning; [TIMP-2]·[IGFBP7], log of product of tissue inhibitor of metalloproteinases-2 and insulin-like growth factor-binding protein 7 concentrations in X units.

Exploratory *post hoc* analyses showed that before the intervention, [TIMP-2]·[IGFBP7] values between the four treatment groups were comparable (Table 3, Fig. 2). After the intervention, patients of the sevoflurane+RIPC group had median [TIMP-2]·[IGFBP7] values of 0.580 (0.230–0.980), which were higher than the propofol+RIPC values of 0.265 (0.130–0.673) and the other two groups (*P*=0.025). A [TIMP-2]·[IGFBP7] concentration of >0.5 ng ml^−2^ 1000^−1^ after RIPC was associated with renoprotection. More patients in the sevoflurane+RIPC group reached a [TIMP-2]·[IGFBP7] concentration of >0.5 ng ml^−2^ 1000^−1^ compared with the propofol+RIPC group (54.2% *vs* 32.8%, *P*=0.019). Conversely, high [TIMP-2]·[IGFBP7] concentrations after cardiac surgery are associated with AKI: 4 h after CPB, [TIMP-2]·[IGFBP7] levels again differed between the four groups (*P*=0.001) (Table 3, Fig. 2). But in contrast to the post-RIPC levels, 4 h after CPB, the concentrations in the sevoflurane+RIPC group were markedly lower, with a median of 0.240, than in any of the other three groups (sevoflurane+sham-RIPC, 0.895; propofol+sham-RIPC, 0.845; propofol+RIPC, 0.735) (Supplementary Table 1). At 12 h after CPB, [TIMP-2]·[IGFBP7] levels did not differ between the groups (Table 3).

**Table 3:** Values of [TIMP-2]·[IGFBP7] at different time points. In each of the four groups, [TIMP-2]·[IGFBP7] showed a significant change over time (*P*_1_<0.0001, *P*_2_<0.0001, *P*_3_=0.0003, *P*_4_<0.0001). The shape of the time course in groups 1, 2, and 3 did not differ significantly (*P*=0.9990). However, group 4 differed significantly from each of groups 1, 2, and 3 (*P*_4__*vs*__1_=0.0047, *P*_4__*vs*__2_<0.0001, *P*_4__*vs*__3_=0.0054). RIPC, remote ischaemic preconditioning; [TIMP-2]·[IGFBP7], log of product of tissue inhibitor of metalloproteinases-2, and insulin-like growth factor-binding protein 7 concentrations in X units. CBP, cardiopulmonary bypass. ∗*P*-values from Kruskal–Wallis test.

Group	1. Pre-RIPC, median (Q1–Q3)	2. Post-RIPC, median (Q1–Q3)	3. 4 h after CPB, median (Q1–Q3)	4. 12 h after CPB, median (Q1–Q3)
Propofol+sham-RIPC (*n*=20)	0.395 (0.153–0.808)	0.265 (0.163–0.555)	0.845 (0.213–1.400)	0.335 (0.213–0.620)
Propofol+RIPC (*n*=60)	0.345 (0.173–0.783)	0.265 (0.130–0.673)	0.735 (0.278–1.450)	0.370 (0.173–0.835)
Sevoflurane+sham-RIPC (*n*=20)	0.370 (0.243–0.683)	0.280 (0.193–0.413)	0.895 (0.340–1.038)	0.400 (0.180–0.810)
Sevoflurane+RIPC (*n*=60)	0.360 (0.160–0.760)	0.580 (0.230–0.980)	0.240 (0.10–0.553)	0.285 (0.190–0.490)
*P*-value∗		0.025	0.001	0.660

### Secondary endpoints

Overall, 62/160 (38.8%) participants developed AKI within 72 h after surgery. Although the AKI rate in the sevoflurane+RIPC group was lower compared with the other groups, it did not reach statistical significance (8/20 [40.0%] propofol+sham-RIPC, 27/60 [45.0%] propofol+RIPC, 9/20 [45.0%] sevoflurane+sham-RIPC, and 18/60 [30.0%] sevoflurane+RIPC; *P*=0.353) (Table 4). All other secondary endpoints showed no significant difference between the groups (Table 4). Frequencies of adverse events were not different between participants receiving RIPC *vs* sham-RIPC, except the development of petechial bleeding, which was more frequent in the two RIPC groups (Table 5).

**Table 4:** Patient-centred outcomes. AKI, acute kidney injury; ICU, intensive care unit; MAKE, major adverse kidney events; RIPC, remote ischaemic preconditioning; RRT, renal replacement therapy. ∗Global *P*-value of the randomised groups propofol+sham-RIPC *vs* propofol+RIPC *vs* sevoflurane+sham-RIPC *vs* sevoflurane+RIPC. ^†^Three patients lost to follow-up at 90 days.

	Propofol+sham-RIPC	Propofol+RIPC	Sevoflurane+sham-RIPC	Sevoflurane+RIPC	*P*-value∗
	(*n*=20)	(*n*=60)	(*n*=20)	(*n*=60)	
Acute kidney injury outcomes, *n* (%)					
AKI within 72 h	8 (40.0)	27 (45.0)	9 (45.0)	18 (30.0)	0.353
Stage of acute kidney injury					
Stage 1	5 (25.0)	17 (28.3)	7 (35.0)	12 (20.0)	
Stage 2	2 (10.0)	8 (13.3)	1 (5.0)	4 (6.7)	
Stage 3	1 (5.0)	2 (3.3)	1 (5.0)	2 (3.3)	
Diagnosis based on					
Creatinine	0 (0.0)	8 (13.3)	1 (5.0)	1 (1.7)	
Urine output	6 (30.0)	13 (21.7)	8 (40.0)	10 (16.7)	
Creatinine and urine output	1 (5.0)	1 (1.7)	0 (0)	7 (11.7)	
RRT	1 (5.0)	1 (1.7)	0 (0)	0 (0)	
Length of stay, median (Q1–Q3) (days)					
ICU length of stay	3.0 (1.3–9.8)	2.0 (1.0–7.0)	3.0 (1.3–5.8)	2.0 (1.0–6.0)	0.863
Hospital length of stay	14.5 (11.0–21.8)	13.0 (10.3–19.8)	12.0 (9.0–19.0)	13.0 (10.0–19.5)	0.646
Outcomes after 30 days, *n* (%)					
Renal recovery at day 30	18 (94.7)	49 (86.0)	19 (100)	51 (89.5)	0.299
RRT at day 30	2 (10.5)	3 (5.3)	1 (5.3)	3 (5.3)	0.843
Mortality at day 30	1 (5.0)	3 (5.0)	1 (5.0)	3 (5.0)	1.000
MAKE_30_	3 (15.0)	12 (20.0)	2 (10.0)	10 (16.7)	0.767
Outcomes after 90 days^†^, *n* (%)					
Renal recovery at day 90	16 (88.9)	48 (87.3)	18 (100)	51 (94.4)	0.287
RRT at day 90	1 (5.6)	2 (3.6)	1 (5.6)	1 (1.9)	0.826
Mortality at day 90	1 (5.3)	4 (6.8)	2 (10.0)	5 (8.5)	0.934
MAKE_90_	3 (15.8)	11 (18.6)	3 (15.0)	9 (15.3)	0.960

**Table 5:** Adverse events. RIPC, remote ischaemic preconditioning.

	Propofol+sham-RIPC	Propofol+RIPC	Sevoflurane+sham-RIPC	Sevoflurane+RIPC
	(*n*=20)	(*n*=60)	(*n*=20)	(*n*=60)
Perioperative myocardial infarction, *n* (%)	0 (0)	1 (1.7)	1 (5.0)	1 (1.7)
Cerebrovascular events/stroke, *n* (%)	0 (0)	3 (5.0)	2 (10.0)	1 (1.7)
Pulmonary embolism, *n* (%)	0 (0)	0 (0)	0 (0)	1 (1.7)
Residual adverse effects on corresponding limb, *n* (%)	0 (0)	0 (0)	0 (0)	0 (0)
Petechial bleeding, *n* (%)	0 (0)	7 (11.7)	0 (0)	12 (20.0)

### High mobility group box protein-1

At baseline, plasma and urinary HMGB-1 levels were similar in the four groups (Supplementary Fig. 1a and b). Immediately after the intervention a significant increase was observed only for the sevoflurane+RIPC group (*P*<0.001) (Supplementary Fig. 1a and b).

## Discussion

Propofol appears to attenuate the RIPC-induced release of HMGB-1 and the two G_1_ cell cycle arrest markers TIMP-2 and IGFBP7 immediately after RIPC. Absolute changes in biomarker levels were higher in the sevoflurane+RIPC group compared with the two sham-RIPC groups and the propofol+RIPC group. As the increase of these biomarkers after RIPC is associated with the renoprotective effects of RIPC, propofol might have a negative influence on the renoprotective effects of RIPC.

RIPC can have protective effects on many organs including the heart and kidneys.^19^, ^20^, ^21^, ^22^ Given its minimal cost and ease of implementation, even if marginally renoprotective it would be of significant clinical importance. However, clinical trials in cardiac surgery have shown conflicting results.^11^^,^^12^ In a single-centre trial of different anaesthetic regimens, a cardioprotective effect of RIPC among patients receiving isoflurane anaesthesia could be demonstrated whereas this effect was not observed in patients receiving propofol.^13^ This was confirmed in a rat model, where the cardioprotective effect of RIPC was abolished in rats anaesthetised with propofol.^14^ The biochemical mechanisms for these differences are unknown.

Cell cycle arrest is a known biological defence mechanism,^22^ and clinical trials have demonstrated an interaction between RIPC and transient increases of cell cycle arrest markers.^9^^,^^23^ The kidney stress markers TIMP-2 and IGFBP7 are both involved in mediating G_1_ cell cycle arrest.^24^ In the RenalRIP trial, patients receiving RIPC showed early and transient increases of [TIMP-2]·[IGFBP7] and elevated levels of HMGB-1 proteins.^9^ Concentrations of [TIMP-2]·[IGFBP7] >0.5 ng ml^−2^ 1000^−1^ after RIPC were associated with a lower incidence of AKI.^9^ The majority of patients in the sevoflurane+RIPC group reached post-RIPC concentrations of >0.5 ng ml^−2^ 1000^−1^, whereas the majority of patients in the other three groups were <0.5 ng ml^−2^ 1000^−1^. Notably, the number of participants reaching this cut-off value was significantly lower in those receiving propofol compared with those receiving sevoflurane. In contrast, [TIMP-2]·[IGFBP7] concentrations >0.5 ng ml^−2^ 1000^−1^ after CPB were associated with AKI in the RenalRIP trial.^9^ Only participants in the sevoflurane+RIPC group stayed below this cut-off after CPB. This biomarker course was recently confirmed in a clinical trial where different doses of RIPC showed similar findings^23^ and in a large clinical trial in China.^25^

The proposed nephroprotective mechanism is based on the release of damage-associated molecular patterns (DAMPs) such as HMGB-1 from remote tissues subjected to periods of ischaemia and reperfusion. These DAMPs are then filtered at the glomerulus, bind to pattern recognition receptors (PRRs) on tubular epithelial cells, which in turn results in renoprotective mechanisms such as transient cell cycle arrest.^26^ The findings in this trial demonstrate that propofol might inhibit this pathway, as the release of HMGB-1 and the subsequent early and transient increase in cell cycle arrest markers was not present in RIPC patients anaesthetised with propofol, but was present in those anaesthetised with sevoflurane. However, other effects might limit the effects of RIPC on organ protection. In a recent trial, RIPC showed no organ-protective effect in patients undergoing major noncardiac surgery although a propofol-free anaesthesia regime was used.^27^ However, the study was not powered to detect a reduction in AKI and the overall rate of AKI was low, indicating a low-risk cohort for postoperative AKI. Future trials are needed to address the molecular mechanisms that link RIPC to transient cell cycle arrest of tubular epithelial cells, and the precise nature of how propofol and other factors interfere with this signalling pathway. In addition, the interaction of RIPC with other interventions is unknown. For instance, infusion of amino acids can reduce the AKI rate after cardiac surgery.^28^ Even though the mechanism of action is unknown and is probably different from RIPC, it is possible that the combination of both interventions has additive effects.

The current study has several strengths. It was a prospective randomised trial in which the investigators were blinded so that the outcome assessment was unbiased. It is the first human study showing that an anaesthetic appears to abolish the renoprotective effect of RIPC. Although [TIMP-2]·[IGFBP7] is only a marker of renal tubular stress, a surrogate for AKI, these biomarkers have very good performance in predicting protection against kidney injury (transient cell cycle arrest) and development of AKI (persistent cell cycle arrest).^9^

Our study also has some limitations. The 75.3% power of the primary statistical analysis is lower than the usual 80%. Although participants in the propofol group did not develop early and transient increases of the cell cycle arrest markers, there were no statistically significant differences in the patient-centred outcomes measured. However, the trial was not powered to detect a difference in AKI rates. One other important limitation of RIPC is that not all patients respond to this intervention.^23^ Therefore, the question arises whether there are pharmacological techniques that can consistently induce a beneficial transient cell cycle arrest in tubular epithelial cells, and subsequently establish a renoprotective effect in all patients. Lastly, the effect of thiopental on the release of HMGB-1, TIMP-2, and IGFBP7 is unclear and could have influenced the results.

In conclusion, consistent with the RenalRIP and the RIPCRenal trials, we found that RIPC in patients anaesthetised with a propofol-free technique leads to an early and transient increase in TIMP-2 and IGFBP7,9,24 whereas this pattern could not be detected in patients receiving propofol.^11^, ^12^ Studies investigating RIPC should not use propofol. Future studies focusing on patient-centred outcomes are needed to confirm the cell cycle-mediated nephroprotective effects of RIPC.

## Authors’ contributions

Conceived and designed the study: all authors

Performed statistical analyses: JG, MM

Acquired data: AZ, HB, LMS, RW, MM

Drafted the manuscript: AZ, HB, LMS, RW, MM

Made critical revision of the manuscript for key intellectual component: JAK

Provided approval for the final version of the manuscript: all authors

## Funding

German Research Foundation (KFO342/1; ME5413/1-1 to MM, ZA428/18-1 to AZ); Deutsche Forschungsgemeinschaft (German Research Foundation; 493624047 [Clinician Scientist CareerS Münster] to HB).

## Declarations of interest

AZ and MM received lecture fees from Biomerieux, Baxter, and FMC. JAK discloses grant support and consulting fees from Biomerieux and Baxter, and is currently a full-time employee of Spectral Medical. AZ and JAK are inventors on patents involving the use of biomarkers together with RIPC held by the Universities of Münster and Pittsburgh together with Biomerieux. The other authors declare that they have no conflicts of interest.

## Figures and Tables

**figs1:**