# Supplementary material for: Impact of propofol or sevoflurane on the renoprotective effect of remote ischaemic preconditioning in cardiac surgery: the HypnoRenalRIP randomised clinical trial
Source: Br J Anaesth. 2025 Sep 22;135(6):1626–34. doi: 10.1016/j.bja.2025.08.055 (PMC12799410; doi:10.1016/j.bja.2025.08.055)
Supplement: Multimedia component 1 [file mmc1.docx]

**Supplement:**

Primary statistical analysis of the primary endpoint

Result of the fitted quantile (median) regression model

- independent variable [TIMP-2]∙[IGFBP7] pre-RIPC: P=0.040
- main effect of the factor RIPC (versus sham-RIPC in reference group Propofol): P=0.755
- main effect of the factor anesthetic agent (sevoflurane versus propofol in reference group Sham-RIPC): P=0.649
- interaction: P=0.077

**Legend Figure 1:** HMGB-1 levels in the plasma **(A)** and in the urine **(B)** before and after the intervention in the four different groups (blue: propofol + sham-RIPC, green: propofol + RIPC, purple: sevoflurane + sham-RIPC, red: sevoflurane + RIPC). The post-RIPC measurements were taken 45 minutes after the intervention. Abbreviations: HMGB1: high mobility group boy protein-1; i.v.: intravenous; ml: milliliter; ng: nanogram; RIPC: remote ischemic preconditioning; [TIMP-2]∙[IGFBP7]: tissue inhibitor of metalloproteinases-2 and insulin-like growth factor-binding protein 7;

**Table 1:** [TIMP-2]∙[IGFBP7] median differences between groups (95% confidence interval)

|  | **Pre-RIPC, median difference* (95% CI)** | **Post-RIPC, median difference* (95% CI), p-value^@^** | **4h after CPB, median difference* (95% CI), p-value^@^** | **12h after CPB, median difference* (95% CI), p-value^@^** |
| --- | --- | --- | --- | --- |
| **Group 1 versus group 2** | -0.01  (-0.18, 0.14) | -0.01  (-0.18, 0.09)  p=0.8151 | -0.04  (-0.39, 0.32)  p=0.8151 | -0.01  (-0.21, 0.12)  p=0.8637 |
| **Group 1 versus group 3** | 0.01  (-0.20, 0.20) | -0.01  (-0.11, 0.11)  p=0.8717 | 0.08  (-0.40, 0.51)  p=0.7169 | -0.03  (-0.27, 0.15)  p=0.7909 |
| **Group 1 versus group 4** | 0.02  (-0.18, 0.16) | -0.19  (-0.50, -0.03)  p=0.0277 | 0.32  (0.03, 0.83)  p=0.0293 | 0.02  (-0.09, 0.18)  p=0.6689 |
| **Group 2 versus group 3** | -0.01  (-0.17, 0.17) | 0.02  (-0.10, 0.18)  p=0.8195 | 0.05  (-0.28, 0.39)  p=0.7109 | -0.01  (-0.22, 0.15)  p=0.8458 |
| **Group 2 versus group 4** | 0.01  (-0.12, 0.13) | -0.14  (-0.33, -0.01)  p=0.0318 | 0.29  (0.13, 0.63)  p=0.0004 | 0.05  (-0.04, 0.17)  p=0.3021 |
| **Group 3 versus group 4** | 0.02  (-0.18, 0.19) | -0.26  (-0.52, -0.05)  p=0.0124 | 0.30  (0.06, 0.72)  p=0.0147 | 0.09  (-0.06, 0.25)  p=0.2552 |

* Median differences were quantified via the Hodges-Lehmann estimator of location shift

@ Mann-Whitney *U* test

Group 1, Propofol + Sham-RIPC; group 2, Propofol + RIPC; group 3, Sevoflurane + Sham-RIPC; group 4, Sevoflurane + RIPC
